# Game of Bones: How Myeloma Manipulates Its Microenvironment

**DOI:** 10.3389/fonc.2020.625199

**Published:** 2021-02-09

**Authors:** Tyler Moser-Katz, Nisha S. Joseph, Madhav V. Dhodapkar, Kelvin P. Lee, Lawrence H. Boise

**Affiliations:** ^1^ Department of Hematology and Medical Oncology, Winship Cancer Institute, Emory University, Atlanta, GA, United States; ^2^ Department of Immunology, Roswell Park Cancer Institute, Buffalo, NY, United States

**Keywords:** multiple myeloma, bone marrow microenviroment, MGUS, smoldering myeloma, myeloma therapy

## Abstract

Multiple myeloma is a clonal disease of long-lived plasma cells and is the second most common hematological cancer behind Non-Hodgkin’s Lymphoma. Malignant transformation of plasma cells imparts the ability to proliferate, causing harmful lesions in patients. In advanced stages myeloma cells become independent of their bone marrow microenvironment and form extramedullary disease. Plasma cells depend on a rich array of signals from neighboring cells within the bone marrow for survival which myeloma cells exploit for growth and proliferation. Recent evidence suggests, however, that both the myeloma cells and the microenvironment have undergone alterations as early as during precursor stages of the disease. There are no current therapies routinely used for treating myeloma in early stages, and while recent therapeutic efforts have improved patients’ median survival, most will eventually relapse. This is due to mutations in myeloma cells that not only allow them to utilize its bone marrow niche but also facilitate autocrine pro-survival signaling loops for further progression. This review will discuss the stages of myeloma cell progression and how myeloma cells progress within and outside of the bone marrow microenvironment.

## Introduction

Multiple myeloma (MM) is defined as a clonal proliferation of malignant plasma cells, and it accounts for roughly 10% of all hematological cancers (1). Myeloma cells retain numerous features of plasma cell biology including a reliance on signals within the bone marrow microenvironment (2). Interestingly, myeloma’s precursor states share the same genetic alterations observed in symptomatic MM patients in both the plasma cells and the microenvironment (3, 4). In advanced stages, myeloma cells can extravasate from the bone marrow leading to extramedullary plasmacytomas and/or circulating plasma cells in the blood (5). Currently, there are ~70 patient-derived myeloma cell lines (HMCL), representing the most advanced stage of myeloma progression whereby myeloma cells survive independently of the bone marrow microenvironment. To this effect, myeloma cells can be compared to an expansive civilization that strategically taps the resources of its niche and when left unchecked will colonize and overtake its host. The malignant cells compete in a “Game of Bones” against the host’s innate defenses and utilize the microenvironment in as a means of gaining an advantage. This review will examine progression of disease from asymptomatic precursor states to MM while shining a light on the changes myeloma cells induce in themselves and within the microenvironment to enable such progression. It will also address the signals that allow myeloma to survive independently of the bone marrow microenvironment in their quest for further growth and expansion.

## Spectrum of Plasma Cell Dyscrasias

### Multiple Myeloma

Historically, establishing the diagnosis of multiple myeloma required both documented bone marrow plasmacytosis (BMPC) ≥10% or an extramedullary plasmacytoma with evidence of end organ damage defined by the CRAB criteria (elevated serum calcium levels, renal insufficiency, anemia, and lytic bone disease). However, in 2014, the International Myeloma Working Group (IMWG) revised the diagnostic criteria to include ultra high-risk patients previously classified as having pre-myeloma or SMM given the ~80% risk of progression to symptomatic disease at two years. These risk factors include BMPC ≥60%, involved to uninvolved serum free light chain ratio >100, and >1 focal lesion on whole body MRI or PET-CT (6–9).

Management and therapy selection for myeloma patients can be determined based on risk stratification. Previously, the International Staging System (ISS) divided disease burden of myeloma into three stages. Serum levels of beta-2 microglobulin (ß2M) and albumin were determined to be the most accurate predictors of disease burden and median survival. Stage I is defined as ß2M < 3.5 mg/L and serum albumin ≥ 3.5 g/dL. In Stage III, ß2M ≥ 5.5 mg/L and serum albumin < 3.5 g/dL. Stage II refers to the intermediate stage of neither Stage I or III (10, 11). Recognizing the important role cytogenetics play in risk stratification, revised ISS (R-ISS) was recently developed still utilizing the ISS, but incorporating both serum lactate dehydrogenase (LDH) levels as well as high risk cytogenetics defined by IgH-MMSET/FGFR3 [t(4,14)] translocations, IgH-MAF [t(14,16)] translocations, and deletion of the p arm of chromosome 17 (del17p) (**Table 1**).

**Table 1:** Defining stages of myeloma.

MGUS				
	Risk Factors	Risk Group	Risk of progression at 20 years (%)	Reference
	(1) Serum M-protein <1.5 g/dL; (2) non-IgG subtype (IgM or IgA); (3) serum FLC ratio <0.26 or >1.65)	Low-risk (0 factors)	5	
		Low-intermediate risk (any 1 abnormal factor)	21	
		High-intermediate risk (any 2 abnormal factors)	37	
		High-risk (all 3 factors abnormal)	58	Rajkumar et al. (Lancet Oncology, 2014)
**SMM**				
*Mayo 2018 model*	Risk Factors	Risk Group	Risk of progression at 2 years (%)	
	(1) M-protein >2 g/dL, (2) BMPC >20%, (3) FLC ratio >20	Low-risk (0 factors)	5	
		Intermediate risk (1 factor)	17	
		High risk (2-3 factors)	46	Lakshman et al. (BCJ, 2018)
*IMWG 2019 model*	(1) M-protein >2 g/dL, (2) BMPC >20%, (3) FLC ratio >20 (4) HR-CTG			
		Low (0 factors)	3.7	
		Low-Intermediate (1 factor)	25	
		Intermediate-High (2 factors)	49	
		High (3+ factors)	72	San Miguel et al. (JCO, 2019)
**MM**				
	Criteria	Stage	Median OS (months)	
*ISS*	B2M<3.5, Alb >3.5	1	62	
	Not meeting criteria for either ISS 1 or 3	2	44	
	B2M >5.5	3	29	Griepp et al. (J. ASCO, 2005)
*R-ISS*	ISS stage 1 without HR-CTG and LDH WNL	1	Not reached	
	Not meeting criteria for either R-ISS 1 or 3	2	83	
	ISS stage 3 with either LDH >ULN and HR-CTG	3	43	Palumbo et al. (J. ASCO, 2015)

MGUS, monoclonal gammopathy of undetermined significance; SMM, smoldering multiple myeloma; MM, multiple myeloma; BMPC, bone marrow plasma cells; OS, overall survival; ISS, International Staging System; R-ISS, revised ISS; B2M, beta2-microglobulin; Alb, Albumin; FLC, free light chain; LDH, lactate dehydrogenase; HR-CTG, high risk cytogenetics; WNL, within normal limit; ULN, upper limit of normal.

Outside of the R-ISS risk stratification model, there are additional favorable and adverse risk factors that aid clinicians in appropriate stratification and subsequent treatment of myeloma patients. These factors include additional cytogenetic features, presence of extramedullary disease, gene expression profiling, and plasma cell proliferation. Standard risk myeloma encompasses 80 to 85% of newly diagnosed myeloma (NDMM) patients and portends good prognosis with a median overall survival (OS) not reached at 10 years (12). Cytogenetic features indicative of standard risk disease includes trisomies of chromosomes 3, 5, 7, 9, 11, 15, 19, and 21 that is referred to as hyperdiploidy as well as IgH-Cyclin D1 [t(11,14)], and IgH-Cyclin D3 [t(6,14)] translocations. High risk patients encompass 15 to 20% of NDMM patients and have median OS of 5 years (13). In addition to high risk cytogenetic features previously described, additional markers of aggressive disease include complex karyotype, defined as three or more changes on standard karyotype analysis. Another marker of high risk disease is amplification of the q arm of chromosome 1 (amp1q). Co-occurrence of 1q gain with other high risk markers portends poor prognosis, and patients with >4 copies of 1q are at very high risk for progression following initial treatment (14). Another marker of high risk includes the least common IgH translocation with MAFB [t(14,20)] which makes up <1% of myeloma patients (13). High risk myeloma patients are also identified by disease burden (ISS stage III), proliferation (BMPC labelling index ≥ 3% *via* thymidine kinase and C-reactive protein (15)), and presence of extramedullary disease (16).

### Multiple Myeloma Precursor Stages

The presence of a precursor state is not known for most NDMM patients as most diagnoses occur at symptomatic stages. However, studies in 2009 from Drs. Michael Kuehl and Ola Landgren used molecular and biological markers to show that myeloma is preceded in virtually all cases by a premalignant state (17, 18). The following two subsections will refer to these precursor states.

### Monoclonal Gammopathy of Undetermined Significance

MGUS was first described in 1961 by Dr. Jan Waldenström who identified a subset of patients with elevated serum and urine immunoglobulin levels without displaying symptoms of malignancy (19). Waldenström labelled this phenomenon a gammopathy, and the term, MGUS, was later coined in 1978 by Dr. Robert Kyle and colleagues (20). The IMWG now defines MGUS as the presence of a serum monoclonal (M) protein or M-protein at <3 g/dL concentration and <10% BMPC with the absence of CRAB criteria (6).

MGUS is found in 3% of Caucasians over the age of 50 and occurs at a 2 to 3-fold higher rate in African Americans (21, 22). Patients diagnosed with MGUS have a 1% risk per year of progressing to symptomatic myeloma, and therefore the standard of care is surveillance without intervention (23). Risk of patient progression can be further stratified using three risk factors: presence of a non-IgG M protein (IgA or IgG), M-protein >1.5 g/dL, and abnormal serum free light-chain (FLC) ratio (24) (**Table 1**). Recently, advancement of technology allowed for detection of precursor cells to MGUS, labelled pre-MGUS (3, 25). As many genomic alterations in MGUS originate in the germinal center, an aberrant clonal population of plasma cells can be formed prior to migration into the bone marrow (26, 27). Furthermore, microenvironment changes present in MGUS have shown to be key regulators in progression to symptomatic stages, and can be targeted in these early stages (3, 28).

### Smoldering Multiple Myeloma

SMM is an intermediate clinical stage in progression between MGUS and multiple myeloma initially described in 1980 after observing a series of six patients with BMPC >10% that continued to have stable disease without treatment for >5 years (29). SMM is defined as the presence of an M-protein at ≥3 g/dL, and/or BMPC percentage of >10% with no evidence of end organ damage defined by the CRAB criteria (hypercalcemia, renal failure, anemia, bone lesions) (30). After the IMWG revised the diagnostic criteria of myeloma, a subset of patients previously classified as having SMM were now reclassified as having symptomatic myeloma. However, this reclassification ultimately only affected a small proportion of SMM patients, and the challenge still remained how to appropriately risk-stratify the remaining patients. SMM is a very heterogeneous disorder encompassing patients that will progress in the first two years and patients with stable low-level disease more than ten years after diagnosis. How then, do we identify which patients are at the highest risk of progression, and how do we safely manage them?

The Mayo 2018 model, also known as the 20/2/20 model, uses three independent risk factors of progression to myeloma: (1) a serum FLC ratio >20, (2) M-protein >2 g/dL, and (3) BMPC >20%. Depending on whether the patient has either 0, 1, or 2–3 of these factors, they are categorized as having either low, intermediate, or high risk SMM corresponding to a 5%, 17%, or 46% risk of progression at 2 years (31). The IMWG validated this model using a retrospective cohort, but added the high-risk cytogenetic features t(4,14), gain(1q), del(17p), and del(13q). Interestingly hyperdiploidy has been shown to be an adverse prognosticator in SMM despite its opposite meaning in MM (32). In this model, SMM patients were grouped into four risk categories (low risk, low-intermediate risk, intermediate risk, high risk) associated with a 2-year progression rate of 3.7%, 25%, 49%, and 72%, respectively (33) (**Table 1**).

Historically, observation was also the standard of care for SMM as with MGUS. However, recently published data has shown the benefit of early intervention with the immunomodulatory agent (IMiD) lenalidomide in high-risk SMM in terms of delaying progression to myeloma (34). The efficacy of using IMiDs in SMM illustrates the role that the microenvironment has in facilitating MM progression. Ongoing clinical trials continue to investigate different therapeutic strategies in SMM, as this continues to be an evolving area of research.

### Extramedullary Multiple Myeloma

Extramedullary multiple myeloma (EMM) refers to hematogenous spread of clonal plasma cell tumors leading to soft tissue tumors at anatomic sites outside the bone marrow (35). This is a separate diagnosis from solitary plasmacytomas which originate from the underlying bone marrow and grow through the cortical bone (36, 37). EMM can present in the liver, skin, central nervous system, pleura, kidneys, lymph nodes, and pancreas and is present in 6%–8% of NDMM cases and 10-30% of relapsed myeloma patients (38, 39). EMM may also present as plasma cell leukemia (PCL), an aggressive variant of the disease with >20% or ≥ 2*10 (9) circulating plasma cells in the blood (40). PCL can either present *de novo*, known as primary PCL, or more commonly as a progression from already diagnosed myeloma, known as secondary PCL.

Extramedullary disease and PCL are considered high risk entities and associated with a poor prognosis with a median OS of less than 6 months (37). Profiling of extramedullary tumors reveals differences from malignant bone marrow plasma cells. Cytogenetics that indicate standard risk myeloma such as hyperdiploidy and t(11,14) are mainly found in BMPC and rarely found in extramedullary plasmacytomas whereas t(4,14) is more commonly seen in EMM (35, 41). However, PCL, while heterogeneous, does have a higher incidence of t(11,14) translocations (40, 42). In relapsed patients, EMM cells undergo a shift from secretion of intact IgG to light chain, and most HMCL secrete only light chain, demonstrating its correlation with myeloma progression (43). The changes in molecular and protein expression that allow myeloma cells to survive and spread outside of the microenvironment will be addressed in a subsequent section of this review.

## The Role of the Bone Marrow Microenvironment

In 1889, Stephen Paget introduced his “seed and soil” hypothesis which postulated that tumor cells (seed) grow preferentially in selective microenvironments (soil) (44, 45). We have seen that plasma cells undergo genomic alterations in the germinal center prior to MGUS (26, 27). Once this clonal population arrives in the bone marrow, it gains access to a wide array of microenvironment signals that facilitate plasma cell survival. Recent studies have found little difference between the microenvironments of MGUS and myeloma, demonstrating that the “soil” has a role in shaping the malignant progression (3, 46). The bone marrow microenvironment produces pro-survival signals for non-malignant long-lived plasma cells, which can live throughout the lifetime of the host, and secrete antibody titers as part of the adaptive immune response (2). Myeloma cells, the aggressive counterparts, use the supportive surrounding stromal cells, osteocytes, and endothelial cells to further their growth. Myeloma precursor states have been shown to mediate progressive growth *in vivo* in humanized mouse models supporting a dominant role for the microenvironment or tumor-extrinsic signals in regulating tumor growth (46). Initial small changes in the microenvironment or molecular changes to myeloma cells themselves cause an expansion of the plasma cell niche throughout the bone marrow.

### Molecular Changes Driving Myeloma Growth

Myeloma cells undergo numerous molecular changes and genetic events which allow proliferation and induce further changes in the bone marrow microenvironment. One family of proteins commonly dysregulated in myeloma are D-type cyclins (47). D-type cyclins are cell cycle proteins that activate cyclin dependent kinase 4 (CDK4) and CDK6, which phosphorylate and inactivate Rb allowing for E2F activation and cell cycle progression (48). Primary genetic translocations such as t(11,14) and t(6,14) directly drive constitutive expression of cyclin D1 and D3 respectively (47, 49, 50). Another translocation t(4,14) which increases the expression of the histone methyltransferase MMSET (NSD2) also indirectly drives activation of cyclin D2 (47, 51). Cyclin D2 can also be dysregulated through t(14,16) and t(14,20) translocations which drive transcription factors that target Cyclin D2 (47). Although infrequent, biallelic inactivation of Rb itself is a subclonal mutation that occurs in 3% of tumors (52). Rb is found on chromosome 13q, and this deletion of this region is the most common mutation in myeloma, frequently accompanying t(4,14), t(14,16), and t(14,20) translocations (53, 54). Recently it was shown that monoallelic deletion of two other genes on 13q which code for Mir15A and Mir16-1 resulted in development of MGUS in wild type C57BL/6 mice and progression of myeloma in the Vk*Myc multiple myeloma mouse model (55).

Myeloma upregulates oncogenes that are typically associated with proliferation in cancer. One such gene is MYC, and its deregulation typically leads to a more aggressive disease phase (56). MYC translocations are found in 15% of human myeloma tumors (57) and include both IgH-MYC translocations [t(8,14)] and IgL-MYC translocations [t(8,22)]. The MYC locus is the most common source of light chain translocations accounting for 40% of these anomalies, and lambda light chain translocations portend a particularly poor outcome compared to kappa light chain translocations (58). Another pathway that is involved in myeloma proliferation is RAS signaling. A secondary mutation that is uncommon in MGUS, KRAS, and NRAS mutations are each found in ~20% of NDMM patients (27, 59). KRAS and NRAS mutations appear to not uniformly activate MAPK signaling pathways and actually lead to distinct downstream transcriptional signatures (60). Interestingly, FGFR3 mutations, which are mutually exclusive with RAS mutations appear to induce MAPK signaling more effectively (60, 61). Finally, the MAPK pathway can be activated by BRAF mutations. BRAF is mutated in 4% of patients with the V600E mutation being the most common (62). Additionally, recent studies have shown a role for cytidine deaminases such as AID and APOBEC in mediating genomic instability in MM cells (63). The expression of these genes, however, is also dependent on interactions with the microenvironment (64).

### Extracellular Matrix

The bone marrow microenvironment provides a layered structure called the extracellular matrix (ECM) which acts as the “home base” for myeloma cells. Homing to the bone marrow is mediated by interaction of myeloma receptor CXCR4 with the chemokine SDF1α (65). This causes a subsequent migration to the stromal compartment of bone marrow. There, it will interact with ECM proteins or other native bone marrow cells.

**Figure 1:**
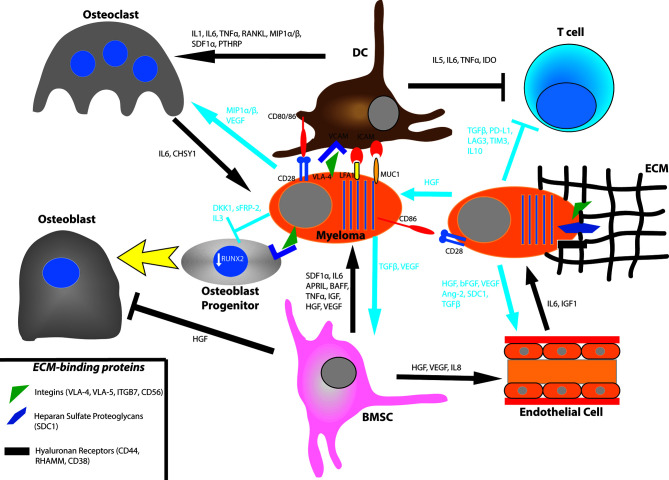
Bone marrow interactions that promote myeloma growth and survival. Myeloma cells bind to the extracellular matrix (ECM) *via* integrins, proteoglycans, and hyaluronan receptors. They also directly bind to bone marrow stromal cell (BMSC) such as dendritic cells (DC) *via* the VCAM-VLA4, ICAM-LFA1, ICAM-MUC1, and CD80/86-CD28 axes. BMSC will produce the cytokines SDF1α, IL6, APRIL, BAFF, TNFα, IGF, HGF, and VEGF. In turn, myeloma cells secrete TGFβ and VEGF for BMSC. Myeloma cells promote osteoclast activation by secreting MIP1α/β and VEGF and by promoting BMSC secretion of IL1, IL6, TNFα, RANKL, MIP1α/β, SDF1, and PTHRP. They also prevent osteoblast differentiation by downregulating RUNX2 *via* direct binding of the VLA-VCAM axis and secretion of DKK1 and IL3. They also secrete sFRP-2 which also suppresses osteoblast differentiation. Osteoclasts produce IL6 and CHSY1 to promote myeloma cell survival. Myeloma cells induce angiogenesis in the bone marrow by secreting HGF, bFGF, VEGF, Ang-2, cleaved syndecan-1 (SDC1), and TGFβ. They also promote BMSC secretion of HGF, VEGF, and IL8. Endothelial cells produce IL6 and IGF1 to influence myeloma cell survival. Myeloma cells promote an immunosuppressive environment by inhibiting T cell function through production of TGFβ, PD-L1, LAG3, TIM3, and IL10. They also signal to BMSC to produce IL5, IL6, TNFα, and IDO.

The ECM consists of proteins such as fibronectin, collagen, osteopontin, hyaluronan, and laminin. Adhesion of myeloma cells has been shown to be important for survival and drug resistance (66, 67). A method of cell-ECM adhesion is activation of integrins, and myeloma cells have shown preference toward very large antigen-4 (VLA-4) aka integrin α4ß1 and integrin ß7 (ITGB7) (68–70). Binding of VLA-4 to fibronectin of the ECM induces activation of nuclear factor κB (NFκB) leading to cell adhesion-mediated drug resistance (CAM-DR) and pro-survival signaling (71). ITGB7 can be regulated by the MAF gene, and as a result, patients with t(14,16) have elevated levels of ITGB7 (51). ITGB7 is necessary for myeloma cell survival and CAM-DR, and has been shown to be constitutively active in myeloma cells (70, 72). Additional integrins such as VLA5 and the beta 5 integrin CD56 play a smaller but active role in myeloma progression (68, 73) (**Figure 1**).

Syndecan-1 or CD138 is a heparan sulfate proteoglycan and a surface marker of myeloma cells. It binds to type I collagen and induces expression of matrix metalloproteinase 1 (MMP1) to promote tumor invasion, bone resorption, and angiogenesis (74, 75). Additionally, syndecan-1 levels on cells correlate with cell survival and growth (76). Heparanase has an intricate interplay with syndecan-1, either causing its clustering and increased adhesion to the ECM or inducing its shedding (77, 78). Soluble syndecan-1 has been shown to promote myeloma tumor growth *in vivo (* 79). Finally, CD44, RHAMM, and CD38 are hyaluronan receptors (67). Hyaluronan is a secreted scaffold protein in the bone marrow. While certain splice variants of CD44 and RHAMM are active in the bone marrow as receptors for hyaluronan and osteopontin, they are generally more involved in extramedullary myeloma. Both proteins regulate the SDF1α/CXCR4 axis, and cell-ECM adhesion with RHAMM is more involved in cell motility (67). CD38 is another hyaluronic acid interacting partner that is expressed in high levels in plasma cells but low levels in other lymphoid and myeloid cells making it an effective target for antibody therapies like daratumumab and isatuximab (80)

### Myeloma–Stroma Cell–Cell Contact

Binding of VLA-4 of a myeloma cell to VCAM of an adjacent stromal cell promotes downstream signaling pathways that activate NFκB and cause cellular survival and proliferation (74). Another myeloma receptor, lymphocyte function-associated antigen 1 (LFA1), will bind to ICAM-1 of an adjacent bone marrow stromal cell (BMSC). LFA1 is an integrin composed of αL and ß2 subunits and is associated with poor prognosis and disease progression in patients as well as increased proliferation in mice (81, 82). Mucin 1 (MUC1) is another transmembrane binding partner of ICAM-1 that has been shown to drive myeloma progression (**Figure 1**). MUC1 will induce proliferation in multiple myeloma by signaling *via* a ß-catenin/TCF4 mechanism to drive MYC gene expression (83).

Plasma and myeloma cells also express CD28, a transmembrane protein classically known for its role in T cell co-stimulation. During this process, MHC of an antigen presenting cell (APC) will first bind to the T cell receptor. The T cell is not fully activated unless CD80/86 of an APC binds to CD28 of a T cell, inducing survival, proliferation, and effector function in T cells (84). Plasma cells retain this CD28 pro-survival signaling capacity, and binding with CD80/86 of a BMSC, e.g., dendritic cell confers survival throughout the lifetime of the host (85, 86). Plasma and myeloma cells are dependent on CD28 signaling through both the PI3K and Vav signaling pathways (87, 88). Knockout of CD28 leads to decreased antibody titers of long-lived plasma cells in mice, and knockdown of CD28 or CD86 with short hairpin RNA leads to myeloma cell death in HMCL (86, 88, 89) (**Figure 1**).

### Myeloma-Bone Marrow Stromal Cell Pro-Survival Cytokines

Multiple myeloma cells also pave the way for their own survival and proliferation by inducing cytokine secretion in BMSC. Direct binding of plasma and myeloma cells to BMSC leads to downstream pathways such as MAPK, NOTCH, and PI3K and cause subsequent transcription and secretion of numerous cytokines. One such cytokine is interleukin-6 (IL6), which has roles in myeloma growth, survival, migration, and drug resistance. IL6 binds to its cognate IL6-receptor (IL6R) and signals through MEK/MAPK, JAK/STAT, and PI3K/Akt pathways (90–92). It also increases dependence on Mcl-1, an anti-apoptotic Bcl-2 protein that is essential for plasma and myeloma cell survival. IL6 upregulates Mcl-1 in a STAT3 dependent manner and induces phosphorylation of Bim, thus increasing affinity of Bim for Mcl-1 over Bcl-2/Bcl-x. This increased binding of the two proteins ultimately leads to stabilization of Mcl-1 (93, 94).

In the absence of IL6, two other cytokines, B-cell activating factor (BAFF) and a proliferation inducing ligand (APRIL) have been shown to have a protective effect on myeloma cells particularly from treatment with corticosteroid (95). BAFF is a member of the tumor necrosis factor (TNF) family and is expressed on the surface of BMSC as well as in a cleaved soluble form. It has been shown to stimulate B cell growth, and, additionally, ligation of its receptors BAFF-R and TACI leads to increased proliferation and survival in myeloma cells (96, 97). APRIL is a secreted protein that will bind to TACI and B-cell maturation antigen (BCMA), a protein which has recently become a target for myeloma CAR-T cell therapy with an 88% response rate (98, 99). BAFF and APRIL-mediated signals also impact survival and growth signals to MM from surrounding dendritic cells (100). They are overexpressed in myeloma cells compared to normal plasma cells illustrating the importance of these cytokines (95). APRIL and BCMA promote cell growth (*via* MAPK and NFκB) and immunosuppression (*via* PD-L1, TGF- ß, and IL10) in myeloma cells (98).

Another member of the TNF family involved in myeloma growth and survival in the bone marrow microenvironment is TNFα. TNFα is a mediator of inflammation and has been found to be significantly higher in supernatants of patients with bone disease than those without (101). While TNFα signaling itself causes a modest increase in proliferation, it induces expression of adhesion molecules resulting in a 2–4 fold increase in binding of myeloma cells to BMSC. It also results in a significant increase in IL6 secretion. Interestingly, TNFα levels decrease with thalidomide treatment which may be a result of downstream effects of the drug’s immunomodulatory effects on bone marrow myeloma cells (102).

Myeloma cells induce BMSC to secrete numerous growth factors. Among them, insulin-like growth factor (IGF) appears to have a sustained and pronounced effect on myeloma proliferation and antiapoptotic signaling. IGF binds to the tyrosine kinase receptor IGF-1R, and additionally influences proteasome and telomerase activities in myeloma cells. IGF is also implicated in drug resistance to cytotoxic chemotherapy, dexamethasone, and proteasome inhibitors (103). It primes myeloma cells to respond to other cytokines and to produce pro-angiogenic cytokines. BMSCs also produce other growth factors such as hepatocyte growth factor (HGF), basic fibroblast growth factor (bFGF) and vascular endothelial growth factor (VEGF) which influence osteoclast activation and angiogenesis (74) (**Figure 1**).

### Osteoclast Interactions

Bone lesions result from osteoclast activation to enable further space for myeloma proliferation in the bone marrow. To directly activate osteoclasts, myeloma cells secrete macrophage inflammatory protein-1α (MIP1α) and MIP1ß. MIP1α binds to C-chemokine receptor 1 (CCR1) and CCR5 while MIP1ß binds to CCR5 and CCR8 to induce osteoclast formation and activity (104–106). MIP1α has been shown to lead to bone destruction, BMSC adhesion, and tumor burden in SCID mice with multiple myeloma (104). In turn, osteoclasts secrete IL6 to stimulate proliferation and growth of not only myeloma cells but other osteoclasts as well (107). Myeloma-osteoclast interaction also upregulates Chondroitin synthase 1 (CHSY1), which induces Notch signaling promoting the survival of myeloma cells (108). Notch signaling, particularly Notch3 and Notch4 stimulation leads to recruitment of osteoclast precursors and increased bone resorption (109, 110).

Interactions between myeloma cells and BMSCs also leads to production of cytokines that stimulate osteoclastogenesis. Binding of VLA4 with VCAM promotes secretion of cytokines such as IL1, IL6, TNFα, and parathyroid hormone related peptide (PTHRP) which promote osteoclast growth (111). Binding of VLA4 and VCAM also lead BMSC to produce receptor activator of NFκB ligand (RANKL). RANKL will bind to its receptor RANK to stimulate osteoclast activation and differentiation and bone lysis (111, 112). RANKL, MIP1α, and IL11 are upregulated by p38 MAPK in BMSCs, and inhibiting p38 MAPK decreases osteoclastogenesis and bone resorption (113) (**Figure 1**). The bone matrix glycoprotein, osteopontin, and the pro-inflammatory cytokine IL17 have also been implicated in osteoclastogenesis and bone resorption. They have been shown to be associated with poor prognosis and osteolytic lesions in patients (114–116).

### Osteoblast Interactions

Myeloma cells also disrupt bone homeostasis by inhibiting osteoblast production and activation. Osteoblasts and BMSC produce osteoprotegerin (OPG) which inhibits the development of bone disease by competing for binding of RANK with RANKL (117). Binding of OPG with RANK prevents osteoclast maturation and activation (118). The ratio between RANKL and OPG is important prognostic indicator in patients and can be influenced in numerous ways (119–121). One way is binding of VLA4 on myeloma cells to VCAM of BMSCs which decreases secretion of OPG and increases secretion of RANKL, thereby tipping the balance in favor of osteoclasts (111, 112). Other factors which augment the RANKL/OPG ratio are activin A and sclerostin (122, 123). Sclerostin is cysteine knot protein which induces apoptosis in osteoblasts and inhibits bone formation (124). Activin A, a member of the TGF- ß superfamily, signals through numerous pathways to promote osteoclast differentiation and is a marker of poor prognosis (122, 125). Interestingly, IL3 can increase osteoclastogenesis by regulating activin A levels (126).

Myeloma cells can also prevent the maturation of osteoblast progenitor cells. Binding of VLA4 of myeloma cells to VCAM of osteoblast progenitors downregulates the activity of RUNX2, a transcription factor that is necessary for the differentiation of osteoblastic cells (127). In addition to increasing the RANKL/OPG ratio, IL7 secretion by BMSC also decreases RUNX2 activity and osteoblast differentiation (119, 127, 128). Recent studies from the Croucher lab have shown that MM-osteoblast interactions may also be important for maintaining dormancy of tumor cells (124)

Secretion of the cytokines Dickkopf 1 (DKK1) and Frizzled related protein 2 (sFRP-2) by myeloma cells contributes to bone resorption as well. DKK1 and sFRP-2 inhibit the canonical Wnt pathway which is responsible for the differentiation of osteoblast progenitor cells (127, 128). DKK1 and sFRP-2 are expressed in multiple myeloma cells of patients with bone lesions. Recombinant DKK1 and sFRP-2 or conditioned media containing either of the two cytokines inhibit differentiation of osteoblast precursor cells *in vitro* and suppress *in vitro* bone mineralization (129, 130). Interestingly, immunodepletion of sFRP-2 led to increased bone restoration suggesting it is necessary for bone resorption. Osteoblast differentiation may take place *via* the bone morphogenic protein 2 (BMP2) pathway. sFRP-2 as well as IL3 inhibit this pathway, thereby stunting osteoblast activation. Additionally, secretion of the cytokines TGF- ß and HGF by BMSC promote osteoclast generation while limiting osteoblast activity (74, 131) (**Figure 1**).

### Endothelial Cell Interactions

Angiogenesis is the creation of new blood vessels through the use of endothelial cells. Patients with progressive myeloma disease show increased level of microvessel density (MVD), a measure of angiogenesis, when compared to those with inactive MGUS (132). This is because myeloma cells crowd the bone marrow microenvironment and generate hypoxic tumors, so they upregulate angiogenesis to deliver oxygen and nutrients while removing catabolites. In the presence of hypoxic conditions, myeloma cells upregulate hypoxia induced factor 1α (HIF1α), which regulates transcription of pro-angiogenic cytokines including HGF, bFGF, VEGF, and Angiopoietin-2 (Ang-2). Myeloma cells may also constitutively produce these cytokines due to genetic mutations or oncogene activation (133).

Adhesion of myeloma to the ECM increases angiogenesis. Expression of adhesion molecules VLA4, LFA1, and CD44 have been shown to correlate with increased angiogenesis in active myeloma (134). Syndecan-1 has been shown to have a prominent role in bone marrow angiogenesis as well. Syndecan-1 is correlated with MVD and facilitates binding of growth factors, particularly HGF, to cells. Not only can syndecan-1 potentiate the surface binding of HGF to cells, but it can also be shed in a soluble form that complexes with HGF to increase potency (135, 136). Myeloma cells also facilitate degradation of the ECM using matrix metalloproteinases (MMP) and heparanase to allow migration of endothelial cells into the surrounding tissue (137, 138).

Myeloma cells stimulate BMSCs to secrete HGF, VEGF, and IL8 to induce neovascularization (139). In turn, endothelial cells will produce IGF1 and IL6 to promote myeloma cell growth. This process can induce an autocrine loop in endothelial cells as they produce VEGF, platelet-derived growth factor (PDGF), Ang-1, HGF, and IL1 to further promote angiogenesis (140) (**Figure 1**).

### Immune Cells

While the previous subsections have addressed allies that myeloma uses to advance itself in the Game of Bones, myeloma cells have an antagonist in the form of antitumor cells in the bone marrow. To overcome this, MM and its precursor MGUS are associated with several alterations in both innate and adaptive immunity. Immune cells increased in MM include regulatory T cells, IL-17-producing T cells, and terminally differentiated effector T cells, however, immunosuppression and exhaustion of these cells was present as early as the MGUS stage (3, 141). The bone marrow increases CD4(+) regulatory T cells and decreases CD4(-)CD8(-) regulatory T cells, and this correlates with increased disease burden (142). Myeloma cells produce proteins such as TGF- ß, PD-L1, LAG3, TIM3, and IL10 that contribute to the immunosuppressive phenotype and T cell anergy (**Figure 1**). Interestingly, these proteins are upregulated in myeloma cells by binding of APRIL to BCMA (98). CD28 ligation with CD80/86 has also been shown to cause BMSC secretion of IL6 and IDO. This occurs *via* “back signaling” of CD80/86 to activate the PI3K pathway. While IL6 normally activates T cells, IDO catabolizes tryptophan in the microenvironment into the toxic metabolite kynurenine. This results in T cell anergy *via* GCN2 kinase-mediated sensing of depleted intracellular tryptophan pools (143, 144). Interestingly, a subset of endothelial cells express low levels of CD80/86 as well as CD40 and ICOS-L in myeloma patients which can trap a population of T cells and stimulate them to induce immunosuppressive proteins (145). Autologous dendritic cells stimulated with tumor antigen can be used to activate T cells *ex vivo* to expand and attack the tumor. Emerging treatments such as targeted antibodies, checkpoint inhibitors and CAR-T cell therapy have aimed to increase the potency of the immune response (28, 141). Currently, advances in mass cytometry and RNA sequencing single cell analyses are being used to identify the immune checkpoint signature of the microenvironment (25). These methods have identified immunosuppressive phenotypes such as regulatory T-cell suppression, secretion of suppressive cytokines and interferons, and increased expression of PD-1 on CD8(+) T and NK cells as early as MGUS (146).

Myeloid derived suppressor cells (MDSC) have been shown to promote immune suppression and angiogenesis in multiple myeloma. They induce myeloma cell survival and proliferation by causing AMPK phosphorylation in myeloma cells. This increases levels of the anti-apoptotic proteins MCL-1 and BCL-2 and the autophagy marker LC3II (147). Myeloma cells in turn will cause an increase of MCL-1 expression and survival in MDSC (148). Another cell type, plasmacytoid dendritic cells (pDC), contribute to immunosuppression of the microenvironment when in direct contact with myeloma cells. While pDC can normally be activated to cause apoptosis of myeloma cells, pDC-myeloma binding *via* E-cadherin can convert pDC into tumor promoting cells (149). Myeloma cells use cell-cell contact to court the pDC to their advantage and signal downstream to inhibit pDC secretion of interferon-α (IFN- α) (149).

Natural killer (NK) cells induce cell death in myeloma cells *via* granzyme and perforin release and other proapoptotic ligands (150). Myeloma cells express CD1d and are also highly sensitive to lysis by NK cells. PD-L1 of a myeloma cell can bind PD-1 of NK cells to suppress their cytotoxic effect of myeloma cells. NK cells are a target of numerous therapies aimed at the immunosuppressive microenvironment. Lenalidomide can be added to checkpoint inhibitors to abrogate this effect and stimulate NK to target myeloma cells (151). The anti-SLAMF7 antibody elotuzumab can also be used to activate NK cells and mediate their activity in myeloma (152). In addition to targeting myeloma cells, the anti-CD38 antibody, daratumumab, also depletes CD38(+) regulatory cells in the bone marrow thus promoting an immune response (153). Recently, daratumumab has been shown to specifically stimulate NK cell activity in myeloma by selectively targeting CD38(+) NK cell populations (154). NK cells and other bone marrow resident immune cells are avenues for immunotherapy and have yielded some initial success in treating myeloma patients at precursor stages (28, 155–158).

## Myeloma Takeover Beyond the Microenvironment

While myeloma cells can be seen circulating in peripheral blood in advanced stages, most EMM is characterized by plasmacytomas in adjacent tissues and organs. Myeloma cells must develop the capacity to extravasate through stroma and ECM into the blood and navigate challenges such as building their own microenvironment in sites outside of the bone marrow. As the disease advances, cells undergo molecular and genomic alterations to promote autocrine loops that facilitate survival and proliferation away from its bone marrow sanctuary. It is notable that although extramedullary growth is a feature of advanced MM, circulating tumor cells can be detected even in early stages of MM (159). This section will explore a new landscape for the Game of Bones and how myeloma cells can undergo changes to survive and expand their niche independently of bone marrow signals.

### Extravasation Model

**Figure 2:**
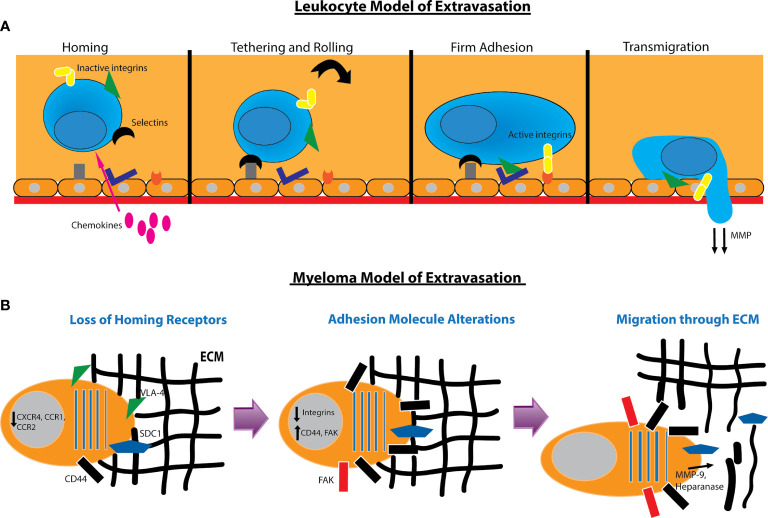
Models for leukocyte and myeloma cell extravasation. **(A)** *Standard leukocyte multistep model of extravasation*. The leukocyte in the bloodstream receives homing signals from chemokines. This is followed by weak adhesion to the endothelium and rolling along the surface. Integrins such as VLA4 and LFA1 are activated to form tight adhesion to the endothelium. The leukocyte then reorganizes its cytoskeleton and degrades the basement membrane to transmigrate through. **(B)** *Model of myeloma extravasation out of the bone marrow.* Myeloma cells downregulate receptors used for homing to the bone marrow. They alter adhesion molecules by downregulating integrins and increasing hyaluronan receptors such as CD44 and RHAMM and expression of focal adhesion kinase (FAK). The myeloma cell will secrete MMP-9 and heparanase as well as induce production of MMP-9 *via* endothelial cells to degrade the extracellular matrix (ECM). Heparanase secretion can cause shedding of SDC1 which also contributes to cell motility. The myeloma cell will then reorganize its cytoskeleton and migrate through the ECM.

While little is known about myeloma extravasation from the bone marrow, we can follow an adaptation of the leukocyte multistep model of extravasation and homing (**Figure 2**). In the standard model, cells first home to an environment as a result of chemoattractants. This is followed by adhesion of the cell to vascular endothelial cells and reorganization of the cytoskeleton to migrate through gaps between these endothelial cells. During this process, the cell degrades basement membrane and extracellular matrix to allow passage until its penetration through (160) (**Figure 2A**). We can reverse this first step for the myeloma cell extravasation model as they must first shed homing signals which tether them to the bone marrow. They must also reduce their affinity to ECM and cells that are specific to the bone marrow and upregulate migratory proteins. Finally, myeloma cells must also degrade the basement membrane to allow passage through gaps created in the bone marrow structure (**Figure 2B**). Once the myeloma cells are in circulation, they may re-enter the vasculature in other marrow compartments *via* the standard leukocyte model of extravasation. They may also form tumors in organs or remain circulating in the blood in the case of PCL.

In order to home to the bone marrow, myeloma cells depend on chemokine signaling. The SDF1α/CXCR4 axis is the myeloma homing pathway most extensively characterized, and impairment of signaling between these molecules is associated with extramedullary transformation (39). Myeloma also depends on CCR1 and CCR2 signaling to regulate migration. Patients with active disease express significantly lower amounts of CXCR4, CCR1, and CCR2 than those with non-active disease, and expression of at least one of these receptors portends favorable clinical outcome (161). Additional chemokine receptors such as CXCR5 and CCR7 are downregulated to promote cell motility and decrease sensitivity to B and T cell cytokines (162).

Myeloma cells alter their adhesion properties to extravasate and migrate through the ECM. EMM plasma cells decrease expression of CD56 while increasing expression of certain CD44 isoforms that are important for proliferation and motility (163). In murine models, decreased expression of P-selectin and VLA4 are associated with increased extramedullary disease (164). Extramedullary myeloma cells also favor focal adhesion kinase (FAK), a protein that mediates an invasive and migratory phenotype. Patients with EMM have significantly increased expression of FAK mRNA compared with patients without extramedullary disease (165). The tetraspanin family of proteins is another family that modulates myeloma adhesion and migration. Two such proteins, CD81 and CD82, are downregulated in HMCL, and exogenous overexpression of these proteins reduces cell motility, invasion, and secretion of MMP-9 (166).

Finally, myeloma cells must degrade the ECM to allow passage through. MMP-9 will degrade basement membrane, and its secretion leads to increased invasion of tumor cells. HMCL have been shown to constitutively secrete MMP-9, and its expression is enhanced by HGF secretion by endothelial cells. Moreover, some HMCL can produce HGF, thus sustaining a loop of increased MMP-9 degradation. Interestingly, SDF1α stimulates MMP-9 production in mouse myeloma model suggesting that SDF1α may have pleiotropic effects in both myeloma cell homing and invasion (167). Myeloma cells also produce heparanase, an enzyme that cleaves heparanase sulfate chains of adhesive proteoglycans such as syndecan-1. Production of heparanase increases motility of myeloma cells and induces a migratory phenotype (168). In part, heparanase and syndecan appear to regulate one another throughout the progression of myeloma and EMM (**Figure 2B**).

### Extramedullary Multiple Myeloma Molecular Changes

Comparison of myeloma cells at extramedullary sites with bone marrow myeloma cells revealed increased subclonal mutations in the extramedullary sites. Morgan et al. proposed a model of myeloma progression that follows the Darwinian mechanism of species evolution. In this model, myeloma cells undergo primary mutations that underlie their growth and expansion. When a bottleneck is applied, subsequent mutations of cells are selected for, thus resulting in surviving subclonal genetic populations (169). In addition to drug treatment, bottlenecks may refer to hypoxia, cell-competition in the microenvironment, and other novel environmental differences in extramedullary sites. Myeloma cells can migrate to other less populated marrow compartments or soft tissue sites and extravasate into these sites or form adjacent plasmacytomas to bone (167).

EMM cells upregulate the adhesion molecules platelet/endothelial cell adhesion molecule-1 (PECAM-1), secreted protein and rich in cysteine (SPARC), and endoglin (ENG), illustrating the shift in adhesion specificity in EMM (170). EMM cells also upregulate nestin, an intermediate filament implicated in metastasis and invasion (171).

A mechanism for myeloma autocrine pro-survival loop lies in the co-expression of CD28 and its ligand CD86. Ligation of CD28 promotes cellular survival and drug resistance in HMCL, and silencing of CD28 and CD86 leads to respective increases in cell death (88, 89). Recent work from our lab has shown that CD86 can signal to confer survival and drug resistance in HMCL. CD86 overexpression induces molecular changes such as increased expression of integrin ß1 and ß7 and interferon regulatory factor 4 (IRF4), a transcription factor necessary for myeloma survival which directly targets MYC (89, 172). Another autocrine loop involved in myeloma survival is secretion of HGF. HMCL and primary myeloma express the tyrosine kinase HGF receptor, c-Met, and produce HGF at variable levels. By this means, EMM cells can signal to stimulate c-Met thus preventing apoptosis and inducing proliferation through autocrine cytokine production (173, 174). Advances in screening technology using CRISPR offer new tools for future elucidation of genes necessary for HMCL survival and proliferation.

### Extramedullary Multiple Myeloma Angiogenesis Signaling

One of the processes highly relied upon in the bone marrow microenvironment, angiogenesis, also has an important role in EMM. Hypoxia in the bone marrow causes myeloma cell upregulation of HIF1α which regulates secretion of proangiogenic cytokines. HIF1α is also upregulated in circulating plasma cells and is associated with myeloma EMT (175). Additional angiogenic factors are upregulated in EMM including VEGF, MMP-9, PECAM-1, and Ang-1. Other angiogenesis related genes such as PDGF, SPARC, NOTCH3, thrombospondin 2, TIMP3, and fibronectin 1 are overexpressed (162). Increased expression of these proteins indicates an important role for angiogenesis in EMM. Although current standard therapies for EMM such as lenalidomide (176) and bortezomib (140) are antiangiogenic, the role of angiogenesis in EMM remains largely unknown.

### Plasma Cell Leukemia Molecular Alterations

PCL is an aggressive variant of EMM marked by rapidly proliferating circulating plasma cells and poor prognosis of patients. Primary immunoglobulin translocations are common in PCL with MAF translocations [t(14,16) and t(14,20)] being the most common followed by t(11,14) and t(4,14). Other common mutations in PCL are MYC translocations which can be found on IgH (5%), IgK(10%), and IgL (10%) loci, respectively (177).

PCL also overexpresses certain genes compared to plasmacytomas including RPL17, CD14, TRAF2, TRAF3, and CCL2. Other affected cancer driver genes include those involved in cell-matrix adhesion and membrane organization (SPTB, CELA1), cell cycle and apoptosis (CIDEC), genome stability (KIF2B), and protein folding (CMYA5). PCL cells are also enriched in functional pathways including Cadherin/Wnt signaling, ECM-receptor, and G2/M cell cycle checkpoint. As PCL are circulating in the blood, there is a downregulation of integrins (CD11a, CD11c, CD29, CD49, CD49e) and other adhesion molecules (CD33, CD117, CD138, CD81) in comparison to EMM. PCL also expresses decreased markers of plasma cells (CD28, CD38) and increased markers of B cells (CD19, CD20, CD45) due to their high prevalence of t(11,14) translocations (177, 178).

## Conclusion

In the Game of Bones, myeloma cells are manipulators that identify allies within the bone marrow microenvironment to exploit and thereby enable their neutralization and evasion of their opposition, the host’s immune defenses. Data that show microenvironment changes as early as MGUS propose that the microenvironment is susceptible to myeloma growth in precursor stages. Mutations in precursor stages beckon a “chicken or the egg” conundrum between myeloma cells and the microenvironment in assessing the advancement of this malignancy. By the time symptomatic myeloma develops, the Game of Bones has already tipped in favor of the cancer. This is because the disease is already quite evolved with numerous means of drug resistance and proliferation in its arsenal, and the cancer has a substantial advantage against innate defenses and chemotherapeutic intervention. In advanced stages, myeloma can readily proliferate in the bone marrow and develop the capacity to transcend the bone marrow.

Recent studies have aimed to tip the advantage back to the side of the host’s defense system, either by effectively targeting myeloma cells and the microenvironment or by strengthening the immune response. As technology and detection tools improve, myeloma cells can be combatted at their early stages before treatment of myeloma or EMM is necessary. Modern genomic approaches such as single cell genomics, mass cytometry, ATAC-seq, whole genome bisulfite sequencing, and integrated phosphoproteomics can elucidate properly tailored treatments for improved efficacy and decreased toxicity of patients. The rise of IMiDs and targeted antibody treatments represent our growing understanding of the therapeutic role of targeting the microenvironment.

Mobilizing the body’s own immune system also improves its odds at winning the Game of Bones. IMiDs, as well as CAR-T cell therapy are of particular interest as they can be utilized to bolster the body’s defenses against its adversary. IMiDs are a potent frontline treatment for MM, and have even been shown to improve patient outcome in the SMM stage (34). Overall, studies of the cancer biology in myeloma cells and their surrounding microenvironment using *ex vivo* patient studies, murine models, and HMCL provide insight to future treatment options and increased efficacy of therapy.

## Author Contributions

TK conceived and wrote the review. NJ, MD, and KL contributed to the writing and editing of manuscript. LB conceived, edited, and oversaw the writing. All authors contributed to the article and approved the submitted version.

## Funding

This work was supported by R01 CA121044 and R01 CA1192844.

## Conflict of Interest

The authors declare that the research was conducted in the absence of any commercial or financial relationships that could be construed as a potential conflict of interest.
